# AI-WAR: a novel warfarin management software with a bidirectional LSTM dosing model improves time in therapeutic range

**DOI:** 10.3389/fphar.2026.1750503

**Published:** 2026-04-29

**Authors:** Yuan Li, Nuo Xu, Long Song, Yaxin Liu, Qi Pei, Yun Kuang, Guoping Yang

**Affiliations:** 1 Center of Clinical Pharmacology, The Third Xiangya Hospital, Central South University, Changsha, China; 2 Xiangya School of Pharmaceutical Sciences, Central South University, Changsha, China; 3 Department of Cardiothoracic Surgery, The Second Xiangya Hospital, Central South University, Changsha, China; 4 Department of Pharmacy, The Third Xiangya Hospital, Central South University, Changsha, China

**Keywords:** anticoagulation management, bi-directional long short-term memory, individualized therapy, time in therapeutic range, warfarin

## Abstract

**Background:**

Warfarin remains the preferred anticoagulant in patients after heart valve replacement. However, its narrow therapeutic window, substantial interindividual variability, and dependence on frequent INR monitoring make effective management challenging. The quality of anticoagulation in real-world practice is often suboptimal, particularly in primary care settings with limited resources.

**Methods:**

We developed an artificial intelligence-based warfarin management system (AI-WAR) integrating remote follow-up, systematic data management, and individualized dosing prediction models. A randomized controlled trial (n = 624) and a prospective registry study (n = 176) were used for model training, while an independent real-world cohort (n = 200) served for external validation. We compared anticoagulation quality and clinical outcomes between conventional management and AI-WAR, and evaluated prediction performance of long short-term memory (LSTM) and bidirectional LSTM (Bi-LSTM) models.

**Results:**

Compared with conventional management, AI-WAR significantly improved median TTR (48.7% vs. 81.3%, P < 0.001), increased time in target INR (36.3% vs. 61.9%, P < 0.001), and reduced both elevated INR percentage (12.0% vs. 16.8%) and overall adverse event rate (9.6% vs. 19.7%, P < 0.001). The Bi-LSTM model exhibited superior dose prediction accuracy (80.3% vs. 66.6%) and achieved 93.2% accuracy for stable dose prediction, while significantly reducing overdose predictions (11.9%, P < 0.001). Subgroup analyses demonstrated robust performance across different genotypes and in scenarios with missing genetic information.

**Conclusion:**

The integration of AI-WAR software and Bi-LSTM prediction models provides an effective and safe decision-support tool for warfarin individualized dosing. This system not only improves anticoagulation quality and reduces adverse events but also shows practical advantages in remote management and primary healthcare settings, supporting broader implementation of precision anticoagulation therapy.

## 1 Introduction

Warfarin, a coumarin-class oral anticoagulant, is widely used for the prevention and treatment of thromboembolic diseases. Although novel oral anticoagulants have increasingly replaced warfarin across many indications, it remains the first-line agent for anticoagulation after heart valve replacement (HVR) owing to its safety and controllability. The prevalence of valvular heart disease is approximately 3.8%, affecting roughly 41 million people, making long-term standardized anticoagulation particularly important (Coffey et al., 2021). Given warfarin’s narrow therapeutic window and substantial interindividual variability (Stein et al., 1998; Heidenreich et al., 2016; Loebstein et al., 2001), over-anticoagulation predisposes to bleeding whereas under-anticoagulation may lead to thrombosis; therefore, regular monitoring of the international normalized ratio (INR) with dose adjustments is required (Hirsh et al., 2001). Genetic polymorphisms are also important contributors to this variability. CYP2C9 is the primary metabolic enzyme of S-warfarin,while VKORC1 encodes vitamin K epoxide reductase protein, the target enzyme of warfarin (Wadelius and Pirmohamed, 2007). Variants in the CYP2C9 and VKORC1 genes are the major genetic determinants of warfarin pharmacokinetics and pharmacodynamics. Studies have demonstrated that polymorphisms in these genes significantly influence warfarin dose requirements and contribute substantially to interindividual variability in anticoagulant response (Wadelius and Pirmohamed, 2007). Consequently, genetic information from CYP2C9 and VKORC1 has been widely incorporated into clinical guidelines for pharmacogenetics-guided warfarin therapy (Johnson et al., 2017). Studies have reported an annual incidence of major bleeding of approximately 0.4%–7.2%, with minor bleeding exceeding 15% (Wadelius and Pirmohamed, 2007; Budnitz et al., 2011).

In recent years, model-based individualized dosing strategies and systematic anticoagulation management have been shown to enhance the effectiveness of warfarin therapy (International Warfarin Pharmacogenetics Consortium et al., 2009; Gage et al., 2008; Zhou et al., 2016; Lafata et al., 2000). Nevertheless, conventional modeling approaches, such as multiple linear regression, Bayesian estimation,and population pharmacokinetic/pharmacodynamic (PK/PD) models, remain limited. Most of these models achieve only modest predictive accuracy (approximately 50%–60%) (Roche-Lima et al., 2019; Hamberg et al., 2014; Zeng et al., 2022) and often demand substantial hardware resources, specialized expertise, and language proficiency, which hinder their broader application in routine clinical practice.

With the rapid advancement of big data analytics and machine learning technologies, novel approaches to anticoagulation management have emerged. Based on high-quality time-series anticoagulation data from 624 patients enrolled in the XY3-WAR study (Guo et al., 2020), our team developed a long short-term memory (LSTM) neural network-based INR prediction model, referred to as LSTM_INR. This model achieved a predictive accuracy of 70.0%, markedly outperforming the conventional maximum *a posteriori* Bayesian model (53.9%) (Kuang et al., 2022). Moreover, it can simulate individualized dosing requirements to achieve target INR values, thereby providing more precise guidance for clinical decision-making (Kuang et al., 2022).

Building upon this model, we developed AI-WAR, a comprehensive warfarin management software (Kuang et al., 2022). AI-WAR integrates professional anticoagulation management functions, including clinician-patient interaction and patient monitoring, with a data management system powered by the LSTM_INR model. Through dynamic analysis of longitudinal treatment data, AI-WAR assists clinicians in optimizing dosing regimens, enabling more precise and effective individualized warfarin therapy.

The present study aims to evaluate the real-world clinical utility of AI-WAR in improving the efficacy and safety of warfarin anticoagulation, to validate the predictive performance of the LSTM_INR model, and to further optimize warfarin dose prediction strategies.

## 2 Materials and methods

### 2.1 Study design and population

The AI-WAR study was designed as a multicenter prospective cohort study (AI-WAR study) conducted at the Second Xiangya Hospital of Central South University and the Third Xiangya Hospital of Central South University, between August 2022 and February 2024. Eligible participants were patients receiving long-term warfarin therapy during this period. Inclusion criteria were: 1) warfarin therapy for at least 3 months; 2) age ≥18 years, with the ability to provide written informed consent and communicate effectively with investigators; 3) willingness to undergo scheduled follow-up visits and either learn to use the AI-WAR software or continue standard medical management; and 4) no history of major bleeding or thromboembolic events within the 3 months prior to warfarin initiation. Exclusion criteria included: 1) missing or incomplete key clinical data; 2) transition to other anticoagulants during the study period.

A total of 400 patients met the eligibility criteria and were enrolled, with a 1:1 allocation to either the AI-WAR intervention group or the control group, based on patient preference. Patients in the intervention group received remote and systematic anticoagulation management via the AI-WAR software. Physicians scheduled follow-up visits according to the patients’conditions. The software reminded patients to test their INR when follow-up was due, and also alerted physicians to the follow-up. It assessed INR levels in real time and provided adjusted dosing recommendations, with physicians referring to these suggestions to preliminarily determine the medication regimen. Patients in the control group received standard warfarin anticoagulation management. After discharge, patients chose hospitals and physicians for INR reexamination based on their individual conditions. Investigators conducted telephone follow-ups every 2 weeks without any additional interventions. Dose adjustments were made entirely based on clinicians’ judgment, without the assistance of artificial intelligence tools. All patients completed a minimum follow-up of 3 months. Baseline data, including demographic characteristics, comorbidities, concomitant medications, baseline INR, and initial warfarin dose, were collected for all participants. In addition, CYP2C9 and VKORC1 genotypes were obtained for patients in the intervention group. After study completion, propensity score matching (PSM) was performed to generate a 1:1 matched cohort to reduce potential selection bias and confounding arising from the non-randomized group allocation, enabling a more balanced comparison of anticoagulation efficacy and safety outcomes. Moreover, longitudinal follow-up data from the 200 patients in the intervention group were used as an external validation set to evaluate the accuracy of the LSTM model in predicting INR values and subsequent dosing requirements.

The study protocol was approved by the Ethics Committees of Xiangya Third Hospital, Central South University (Approval No. R23006), and Xiangya Second Hospital (Approval No. 2023 Ethics [Research] No.148), and was registered at the Chinese Clinical Trial Registry (Registration No. ChiCTR2300071005). Written informed consent was obtained from all enrolled patients.

### 2.2 Outcome measures

The primary efficacy endpoint of this study was the percentage of time in therapeutic range (%TTR) (Ng et al., 2020), and TTR was calculated using the Rosendaal method, which assumes linear change in INR between two consecutive measurements (Rosendaal et al., 1993). The secondary efficacy endpoint was the percentage of international normalized ratio (INR) measurements within the therapeutic range. The INR target ranges for the remaining patients were determined by indication and comorbidities. For anticoagulation therapy, the target INR range was 2.0–2.5 after mechanical valve replacement, 1.8–2.3 after bioprosthetic valve replacement, and 1.5–2.5 after valve repair (Xu et al., 2016). The safety outcome indicators were the percentage of INR measurements exceeding the therapeutic range and the incidence of bleeding events. Bleeding events were classified into major bleeding and non-major bleeding. Major bleeding was defined as any clinically overt bleeding associated with a fall in hemoglobin of 2 g/dL or more, or that required transfusion or surgical intervention. Non-major bleeding included any clinically overt bleeding that did not meet the criteria for major bleeding. All safety events were adjudicated by a blinded independent committee, which reviewed all reported adverse events to ensure consistency and accurate classification.

To assess the predictive performance of the LSTM model, the following criteria were established: the proportion of predicted INR values within ±30% of observed values, defined as INR prediction accuracy; the proportion of predicted doses within ±20% of the actual prescribed doses, defined as dose prediction accuracy.

In addition, dose stabilization was defined as follows: within 7 days after initial dosing, if the warfarin dose remained unchanged for 14 consecutive days while INR values consistently remained within the therapeutic range, the corresponding dose was defined as the stable dose. The proportion of predicted doses within ±20% of this stable dose was defined as stable dose prediction accuracy.

### 2.3 Modeling data

The model training datasets were derived from two previously completed studies: a randomized controlled trial (XY3-WAR, n = 624; ClinicalTrials.gov registration No. NCT02211326) and a prospective registry study (n = 176; Chinese Clinical Trial Registry No. ChiCTR2100052089). The intervention group data from the present AI-WAR study were used as an external validation set. All data were standardized and converted into structured text format, with feature values normalized according to Formula 1.

$$x_{norm}=\frac{x_{i}-\bar{x}}{x_{std}}$$ (1)

Model variables were categorized into two groups. Fixed variables included age, height, weight, CYP2C9 and VKORC1 genotypes, and concomitant use of amiodarone or digoxin (Lee et al., 2002; Tyson et al., 2018; Dumond et al., 2010). Time-series variables included the adjusted warfarin dose at the previous visit (Dose_i-1_), the INR value at the current visit (INRᵢ), the interval in days between the current and next visits (Interval_i+1_), and the adjusted dose at the current visit (Doseᵢ). The output of the dose prediction model was defined as the warfarin dose required at the next visit to achieve the target INR range.

### 2.4 Dose prediction model

**FIGURE 1:**
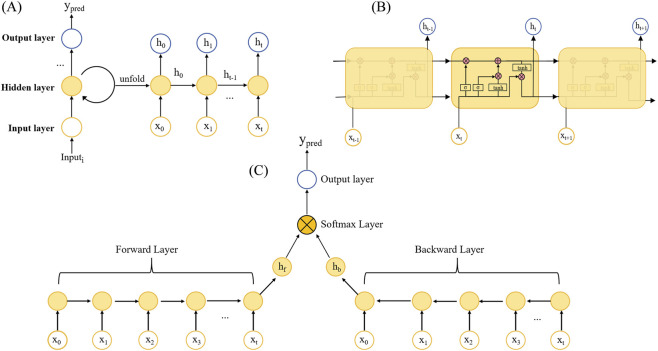
Model demonstration diagram. **(A)** The overall workflow of model process. **(B)** Internal structure of LSTM hidden layer. **(C)** Basic structure diagram of Bi-LSTM.

A bidirectional long short-term memory (Bi-LSTM) network was employed as the modeling approach for dose prediction. Bi-LSTM is an advanced recurrent neural network (RNN) architecture that effectively captures both short- and long-term dependencies in sequential data, simultaneously processing information in forward and backward directions (Kwak et al., 2021; Cheng et al., 2022; Lilhore et al., 2024). Unlike conventional unidirectional RNNs, Bi-LSTM incorporates the characteristics of backpropagation neural networks (BPNNs), enabling input sequences to be processed by two independent LSTM layers, one moving forward and the other backward. This bidirectional mechanism facilitates feature extraction from both directions, and the hidden states are subsequently concatenated or combined to generate a comprehensive bidirectional representation with full contextual information. The structural diagram of the model is presented in (Figure 1C).

During model development, structured data were input into the Bi-LSTM network for training. For comparative purposes, a unidirectional LSTM model was constructed as a control. The overall workflow of the modeling process is illustrated in (Figure 1A). Fixed variables were processed using a feedforward neural network (FFN), whereas time-series variables were handled by either the Bi-LSTM or LSTM network (Figure 1B). The most informative features were extracted via max pooling. Finally, the processed outputs of the two variable types were concatenated along the feature dimension to generate the final prediction output.

### 2.5 Model training and validation

The mean squared error (MSE) was selected as the loss function (Formula 2). Model training was performed using ten-fold cross-validation. Of the dataset, 10% was randomly allocated as the test set, while the remaining 90% was further divided into a training set (90%) and a validation set (10%). To prevent overfitting, early stopping was applied during training, with the maximum number of iterations set to 100. Training was terminated if no significant reduction in loss was observed for 10 consecutive epochs. Initial model performance was evaluated by comparing predicted doses with actual doses.

$$loss=\frac{\sum_{i=1}^{n}{\left(y_{i,pred}-y_{i}\right)}^{2}}{n}$$ (2)

Hyperparameter optimization was conducted sequentially, focusing on the number of hidden units, batch size, number of LSTM layers, and learning rate. For each hyperparameter configuration, the model was trained three times, and the mean predictive accuracy on the test set was used to evaluate its suitability. The optimized hyperparameters were a hidden layer size of 128, a batch size of 32, two LSTM layers, and a learning rate of 0.025. Using a stepwise optimization strategy, the optimal values for each hyperparameter were determined iteratively until an optimized configuration was obtained.

Once finalized, the optimized model was trained ten times, yielding ten candidate models. Model performance and robustness were comprehensively evaluated based on MSE, mean absolute error (MAE), root mean square error (RMSE), and coefficient of determination (*R* ^2^). The candidate model with the best overall predictive performance was thus selected as the final model. The predictive accuracy of the Bi-LSTM model was statistically compared with that of the unidirectional LSTM model using the chi-square test.

### 2.6 Statistical analysis

Data preprocessing was performed using Microsoft Excel, while statistical analyses were conducted with SPSS version 28.0 (IBM Corp., Armonk, NY, USA) and R version 4.2.1 (R Foundation for Statistical Computing, Vienna, Austria). A two-sided P value < 0.05 was considered statistically significant. Continuous variables with normal distribution were expressed as mean ± standard deviation and compared using independent-samples t tests. Non-normally distributed continuous variables were expressed as median (interquartile range) and compared using the Wilcoxon rank-sum test. Categorical variables were summarized as frequencies (percentages) and compared using the chi-square test or Fisher’s exact test as appropriate.

**FIGURE 2:**
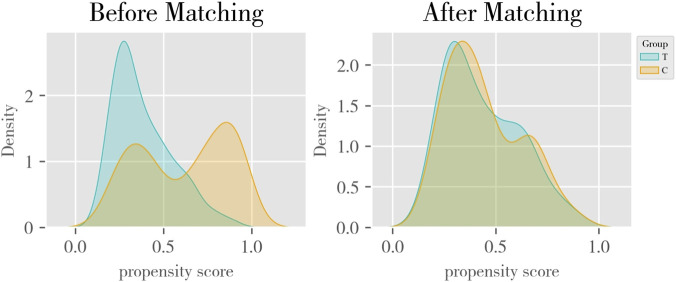
Propensity score probability density curves before and after PSM.

PSM was performed using logistic regression in R to estimate propensity scores. Matching covariates included age, sex, height, weight, indications for warfarin therapy, and comorbidities. A caliper width of 0.1 was applied (Schober et al., 2021), and patients were matched 1:1 using the nearest-neighbor method without replacement. Standardized mean differences (SMD) were calculated for all covariates to assess balance, with SMD <0.10 considered indicative of good balance. After matching, baseline characteristics between groups were re-compared using the same statistical methods. All hypothesis tests were two-sided (α = 0.05). Continuous variables were compared using independent samples t-test (normal distribution) or Mann–Whitney U test (non-normal distribution). Categorical variables were compared using Pearson χ^2^ test or Fisher’s exact test (unordered) or Mann–Whitney U test (ordered). A *P* value > 0.05 was considered indicative of adequate balance between matched groups, and the matching effect was evaluated using the propensity score probability density curve (Figure 2).

## 3 Results

### 3.1 Participants

Baseline characteristics of the 400 enrolled patients are presented in Table 1, with 200 assigned to the intervention group and 200 to the control group. Before propensity score matching (PSM), the control group was slightly older than the intervention group (56.3 vs. 55.7 years), though the difference was not statistically significant (*P* = 0.583). The proportion of females was lower in the control group (45% vs. 55%, *P* = 0.046), while mean height (162.3 cm vs. 160.7 cm, *P* = 0.042) and body weight (60.5 kg vs. 58.8 kg, *P* = 0.102) were higher. Significant differences were also observed between the two groups in terms of treatment indications, comorbidities, and concomitant medications. After PSM, all baseline characteristics were well balanced between the two groups, with no statistically significant differences (*P* > 0.05).

**TABLE 1:** Demographic and clinical informationographic and clinical information.

Characteristics	Before matching		Statistics	*P*	SMD	After matching		Statistics	*P*	SMD
	Test group (n = 200)	Control Group (n = 200)				Test group (n = 118)	Control Group (n = 118)			
Age (y)	55.7 ± 10.3	56.3 ± 11.2	*t* = −0.549^1^	0.583	0.055	53.8 ± 10.4	53.4 ± 10.0	*t* = 0.300^1^	0.764	0.039
Gender, n (%)	​	​	χ^2^ = 4.000^2^	0.046	0.201	​	​	χ^2^ = 1.088^2^	0.297	0.136
Female	110 (55.0)	90 (45.0)	​	​	​	60 (50.8)	52 (44.1)	​	​	​
Male	90 (45.0)	110 (55.0)	​	​	​	58 (49.2)	66 (55.9)	​	​	​
Height (cm)	160.7 ± 8.2	162.3 ± 7.9	*t* = −2.043^1^	0.042	0.205	161.9 ± 8.5	162.4 ± 8.0	*t* = −0.404^1^	0.686	0.053
Weight (kg)	58.8 ± 11.0	60.5 ± 10.2	*t* = −1.641^1^	0.102	0.165	60.2 ± 11.4	61.0 ± 9.7	*t* = −0.547^1^	0.585	0.071
Indication, n (%)										
Biological valve replacement	94 (47.0)	64 (32.0)	χ^2^ = 9.415^2^	0.002	0.311	47 (39.8)	50 (42.4)	χ^2^ = 0.158^2^	0.691	0.052
Mechanical valve replacement	57 (28.5)	79 (39.5)	χ^2^ = 5.392^2^	0.020	0.234	41 (34.7)	44 (37.3)	χ^2^ = 0.165^2^	0.684	0.053
Prosthoplasty	173 (86.5)	120 (60.0)	χ^2^ = 35.839^2^	<0.001	0.627	97 (82.2)	96 (81.4)	χ^2^ = 0.028^2^	0.866	0.022
Other thrombotic diseases	7 (3.5)	32 (16.0)	χ^2^ = 17.757^2^	<0.001	0.431	5 (4.2)	4 (3.4)	χ^2^ = 0.000^3^	1.000	0.044
Concomitant disease/medication, n (%)										
Hypertension	46 (23.0)	74 (37.0)	χ^2^ = 9.333^2^	0.002	0.309	31 (26.3)	31 (26.3)	χ^2^ = 0.000^2^	1.000	0.000
Diabetes	12 (6.0)	17 (8.5)	χ^2^ = 0.929^2^	0.335	0.097	7 (5.9)	6 (5.1)	χ^2^ = 0.081^2^	0.775	0.037
Amiodarone	6 (3.0)	13 (6.5)	χ^2^ = 2.708^2^	0.100	0.165	3 (2.5)	6 (5.1)	χ^2^ = 0.462^3^	0.497	0.133
Digoxin	176 (88.0)	99 (49.5)	χ^2^ = 68.992^2^	<0.001	0.913	97 (82.2)	98 (83.1)	χ^2^ = 0.030^2^	0.864	0.022
Therapeutic efficacy and safety										
%TTR	76.1 ± 18.4	45.8 ± 27.5	​	<0.001	​	78.0 ± 16.4	48.5 ± 25.8	​	<0.001	​
Number of INR measurements	1939	1987	​	​	​	1,210	1,223	​	​	​
INR in range	1,186 (61.2)	710 (35.7)	​	<0.001	​	749 (61.9)	444 (36.3)	​	<0.001	​
Excessive INR	255 (13.2)	332 (16.7)	​	0.002	​	145 (12.0)	206 (16.8)	​	<0.001	​
Adverse events	27 (13.5)	57 (28.5)	​	<0.001	​	18 (9.6)	37 (19.7)	​	<0.001	​

^1^Independent samples t-test.

^2^Pearson χ^2^ test.

Supplementary Table S1 summarizes demographic and clinical characteristics across the three clinical studies. The randomized controlled trial (RCT, XY3-WAR) and registration study were a previously completed study, whereas the AI-WAR study represents the present prospective cohort study. In the randomized controlled trial (RCT), the primary indications were atrial fibrillation (AF) and deep vein thrombosis (DVT), with a mean baseline INR of 1.0. In contrast, valvular heart disease (VHD) was the predominant indication in both the registry study and the AI-WAR study, with mean baseline INRs of 1.4 and 1.5, respectively. Regarding comorbidities, hypertension and diabetes were more prevalent in the RCT cohort (51.1% and 33.2%) compared with the registry (20.5% and 5.7%) and AI-WAR (23.0% and 6.0%) cohorts. With respect to concomitant medication, only 16.3% of patients in the RCT used digoxin, whereas the proportions were substantially higher in the registry (84.7%) and AI-WAR (88.0%) cohorts. The distribution of VKORC1 and CYP2C9 genotypes in all three studies was consistent with those reported in the general Han Chinese population.

During data processing, 36 patients who withdrew on days 4 or 5 after enrollment were excluded from the RCT dataset, resulting in 4,578 structured records from 624 patients. In the registry study, patients with missing baseline INR values or fewer than three follow-up visits were excluded, leaving 176 patients contributing 709 valid records. In total, the modeling dataset comprised 5,287 records from 800 patients, while the external validation dataset included 1741 records from 200 patients.

### 3.2 Anticoagulation outcomes

As shown in Table 1, after PSM, the intervention group had a significantly higher median TTR compared with the control group (81.3% vs. 48.7%, *P* < 0.001). The proportion of INR values within the therapeutic range was also higher (61.9% vs. 36.3%, *P* < 0.001). Regarding safety outcomes, the intervention group had significantly lower rates of supratherapeutic INR (12.0% vs. 16.8%, *P* < 0.001) and overall adverse events (9.6% vs. 19.7%, *P* < 0.001). Supplementary Table S2 details the incidence of specific adverse events during follow-up. Both before and after PSM, the number of patients experiencing each category of adverse events was consistently lower in the intervention group. Moreover, the overall incidence remained significantly reduced compared with controls.

**FIGURE 3:**
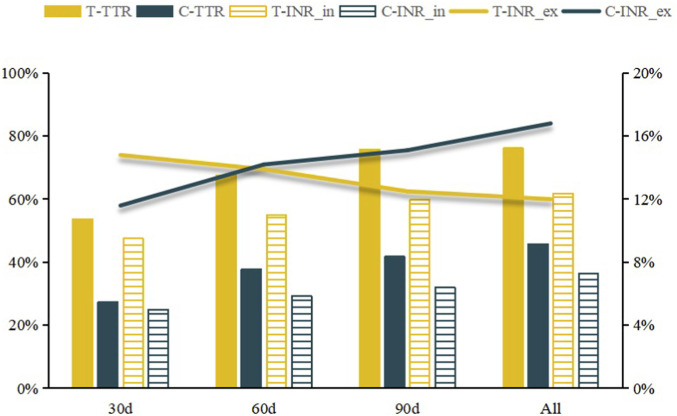
Anticoagulation Therapy Indicator Comparison Chart (The left Y-axis represents percentage values, and the right Y-axis represents ratio values).

To further evaluate longitudinal changes in anticoagulation control, follow-up was stratified into three time intervals: 0–30 days, 0–60 days, and 0–90 days. As shown in Figure 3, the intervention group consistently demonstrated significantly higher median TTR and proportions of INR values within the therapeutic range across all intervals (*P* < 0.001 for all comparisons). The proportion of supratherapeutic INR values declined progressively in the intervention group, whereas it increased in the control group. At 0–30 days, no significant difference was observed between groups (14.8% vs. 11.6%, *P* = 0.074). By 0–60 days, the rates were nearly identical (13.9% vs. 14.2%, *P* = 0.878). However, with longer treatment duration, the between-group difference widened, and over the full follow-up period the intervention group maintained a significantly lower proportion of supratherapeutic INR values (12.0% vs. 16.8%, *P* < 0.001).

### 3.3 LSTM model evaluation

Among the 1,741 dose adjustment records from 200 patients in the intervention group, the LSTM model achieved a prediction accuracy of 66.6% for actual warfarin dosing, with 1,160 correct predictions, 485 overpredictions (27.9%), and 96 underpredictions (5.5%). For INR prediction, the accuracy reached 80.2%, with 1,397 correct predictions, 146 overpredictions (11.0%), and 207 underpredictions (8.7%).

During follow-up, 178 of the 200 patients in the intervention group achieved a stable dosing period, with an average stable dose of 2.59 mg (variance = 0.85). A total of 657 stable dose records were used to test the model. Under these conditions, the LSTM model demonstrated higher predictive accuracy, with 470 correct predictions (71.5%), 184 overpredictions (28.0%), and only three underpredictions (0.5%).

Overall, both in INR and dose prediction, the overprediction rate of the LSTM model was significantly higher than the underprediction rate.

### 3.4 Bi-LSTM dose model

Following hyperparameter tuning, the final Bi-LSTM model was configured with a hidden layer size of 128, a batch size of 32, two LSTM layers, and a learning rate of 0.025. As shown in the Supplementary Figure S1, the loss function consistently converged across all ten training iterations. On the test set, the Bi-LSTM dose prediction model achieved an average accuracy of 80.1% and an average *R* ^2^ of 0.719. The small standard deviations and coefficients of variation across performance metrics indicate good model stability (Supplementary Table S3).

In external validation, the Bi-LSTM model achieved an average prediction accuracy of 79.1% for actual dosing and 90.7% during the stable dosing period. Among the ten models, the tenth iteration performed best, with an accuracy of 80.3% for actual dosing and 93.2% for stable dosing, and was therefore selected as the final model (Supplementary Table S4).

### 3.5 Model performance comparison

As a comparator, a re-trained LSTM model was evaluated across ten training iterations (Supplementary Figure S2; Supplementary Table S5). In external validation, the LSTM model achieved an average prediction accuracy of 71.1% for actual dosing and 80.6% during the stable dosing period. The first LSTM model showed the best performance, with accuracies of 72.4% and 84.0%, respectively, and was selected as the final comparator model.

**FIGURE 4:**
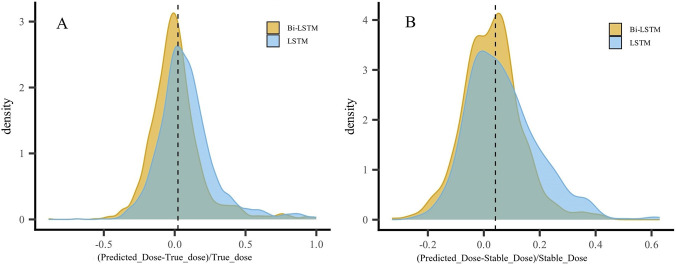
Prediction error density plot. **(A)** Error density plots for predicted actual dosages by the Bi-LSTM model and the LSTM model. **(B)** Error density plots for predicted stable dosages by the Bi-LSTM model and the LSTM model.

Supplementary Table S6 summarizes the comparative results of the two models. Although the Bi-LSTM model still exhibited a higher overprediction than underprediction rate, it was substantially lower than that of the LSTM model. As shown in the Figure 4, prediction errors of the Bi-LSTM model were more concentrated, particularly around zero error, resembling a normal distribution. In contrast, the LSTM model displayed a clear negative skew, indicating a systematic tendency to overestimate the actual dose.

To further enhance the clinical interpretability of model performance, we additionally evaluated the absolute dose error (mg/day) between predicted and reference doses. As shown in Supplementary Table S7, the Bi-LSTM model consistently demonstrated lower absolute dose errors than the LSTM model for both true dose and stable dose. Moreover, stratified analyses across clinically relevant dose ranges (<2 mg/day, 2–3 mg/day, and >3 mg/day) showed that the Bi-LSTM model maintained superior performance across all dose groups. These findings further support the robustness and clinical applicability of the Bi-LSTM model.

**FIGURE 5:**
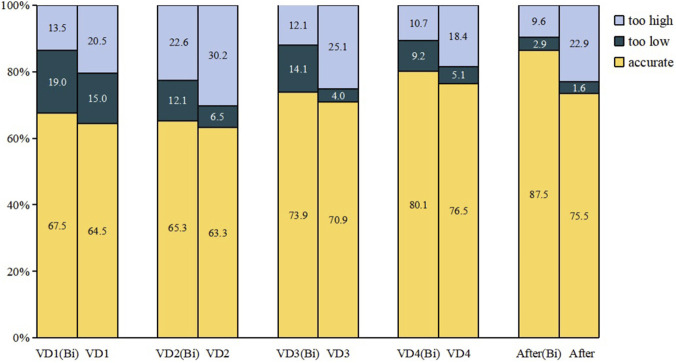
Model performance comparison chart.

Figure 5 presents the prediction accuracies of the two models across follow-up visits. With increasing visits, the Bi-LSTM model achieved accuracies of 67.5% (visit 1), 65.3% (visit 2), 73.9% (visit 3), 80.1% (visit 4), and 87.5% (visit 5). During the first four visits, the Bi-LSTM model showed slightly higher accuracy than the LSTM model, though differences were not statistically significant. From the fourth visit onward, the Bi-LSTM model demonstrated significantly better prediction accuracy (*P* < 0.05).

### 3.6 Subgroup analysis

Patients were stratified into normal response, sensitive response, and highly sensitive subgroups based on CYP2C9 and VKORC1 genotypes (Supplementary Table S8). Compared with the normal response subgroup, both models showed decreased performance in the sensitive subgroup. For the Bi-LSTM model, the accuracy of actual dose prediction declined from 80.8% to 76.2% (*P* = 0.129), and that of stable dose prediction from 93.2% to 92.9% (*P* = 0.909). In contrast, the LSTM model showed a significant reduction in accuracy, with actual dose prediction falling from 73.9% to 63.2% (*P* = 0.002) and stable dose prediction dropping from 88.5% to 53.6% (*P* < 0.001). Moreover, the overprediction rate of the LSTM model increased significantly in the sensitive subgroup (all *P* < 0.001), indicating reduced safety in its predictions.

### 3.7 Impact of genotype information

The impact of missing genotype information on model performance is summarized in Supplementary Table S9. When genotype data were unavailable, the Bi-LSTM model’s accuracy for actual dose prediction decreased to 78.7% (*P* = 0.240), and its accuracy for stable dose prediction declined to 90.1% (*P* = 0.046), accompanied by an increase in the overprediction rate to 9.6% (*P* = 0.007). By contrast, missing genotype information had a more pronounced effect on the LSTM model, reducing its accuracy for actual dose prediction to 68.6% (*P* = 0.013) and increasing the overprediction rate to 28.0% (*P* = 0.001). Its accuracy for stable dose prediction decreased to 73.7% (*P* < 0.001), while the overprediction rate increased to 25.7% (*P* < 0.001).

## 4 Discussion

Warfarin remains a cornerstone of anticoagulation therapy despite its narrow therapeutic window and considerable interindividual dose variability, both of which pose major challenges in clinical practice. Patients on warfarin require regular coagulation monitoring and dose adjustments based on test results. Although novel oral anticoagulants (NOACs), such as rivaroxaban and dabigatran, offer greater convenience, warfarin continues to be the first-line therapy for patients undergoing cardiac valve surgery due to its well-established safety profile in this population (Writing Group Members et al., 2019; Vahanian et al., 2022; Eikelboom et al., 2013). The quality of anticoagulation is commonly evaluated by the percentage of time in therapeutic range (TTR), with TTR ≥65% generally considered adequate. In this study, the control group achieved a median TTR of only 48.7%, falling below the acceptable threshold. This finding is consistent with previously reported data from Chinese atrial fibrillation patients, where the average TTR was approximately 47%, highlighting the suboptimal quality of warfarin anticoagulation under conventional management practices.

Several factors may account for this inadequate anticoagulation quality. First, limitations in outpatient time and medical resources often prevent physicians from providing systematic and continuous dose adjustment guidance for every patient. Second, the long-term requirement for frequent INR monitoring reduces patient adherence, with some patients either skipping follow-up visits or failing to take medication as prescribed. Third, anticoagulation management in most hospitals in China—particularly in primary care institutions—remains underdeveloped, and clinicians may have limited experience in dose adjustment based on INR values. Previous studies have reported an annual incidence of major bleeding ranging from 0.4% to 7.2% and minor bleeding up to 15% in patients on warfarin, underscoring the need for improved effectiveness and safety in routine warfarin management (Johnson et al., 2017). Finally, genotype information (CYP2C9 and VKORC1) was available only in the intervention group, whereas the control group received standard care without pharmacogenomic testing. This design reflects the primary objective of the study, which was to evaluate the clinical utility of AI-WAR as an integrated anticoagulation management system that incorporates genotype-informed dose prediction and patient management. In this context, genotype information was used as one of the input variables for the LSTM-based dose prediction model embedded within the AI-WAR system, rather than as an independent clinical intervention. Therefore, the observed improvements in the intervention group should be interpreted as the combined effect of the embedded LSTM model—integrating both genetic and clinical information—and the patient management functions of the AI-WAR system.

In this study, patients managed with the AI-WAR software demonstrated significant improvements in anticoagulation outcomes: the median TTR increased from 48.7% to 81.3% (*P* < 0.001), the percentage of ideal INR values improved from 36.3% to 61.9% (*P* < 0.001), while both the percentage of supratherapeutic INR values (12.0% vs. 16.8%) and the overall incidence of adverse events (9.6% vs. 19.7%) were significantly lower than those of the control group (both *P* < 0.001). These findings indicate that AI-WAR-based remote anticoagulation management can optimize therapeutic efficacy and enhance treatment safety. Importantly, the system enables a single pharmacist to manage multiple patients simultaneously in a structured manner, thereby improving postoperative recovery, enhancing medication adherence, and reducing the incidence of warfarin-related adverse events and hospital readmissions. AI-WAR has been validated as a safe and effective tool for managing personalized warfarin anticoagulation. However,the integration of AI-WAR into the intervention cohort inherently altered the data-generating process. Patients in the intervention group received more frequent monitoring and real-time dosing recommendations, which likely influenced the variability of INR measurements and dosing adjustments compared to standard care. This may also contribute to the improvement in TTR.

Compared with traditional anticoagulation clinics or manual follow-up systems, AI-WAR offers several key advantages: 1) It assists physicians in efficiently managing patient data and follow-up schedules, thereby improving clinical workflow; 2) It supports remote management by allowing patients to complete INR testing at nearby healthcare facilities based on their specific circumstances. This reduces both time and financial burdens, thereby facilitating increased testing frequency and improved treatment adherence; 3) It eliminates geographical barriers, enabling patients in remote areas to access professional anticoagulation management services; 4) It automatically integrates follow-up data into a continuous, transferable, and visualized electronic medical record, minimizing information loss and facilitating long-term tracking; 5) By integrating the LSTM-based dosing prediction model, it reduces reliance on individual physician experience and enhances confidence and consistency in warfarin dosing decisions.

In our preliminary studies, we identified a key limitation of conventional models whose predictions rely on factors measured at a single time point, typically during either the initiation or maintenance phase of therapy (Asiimwe et al., 2021; Steiner et al., 2021). However, warfarin therapy often requires long-term or even lifelong administration, and its dose-response relationship exhibits marked temporal dynamics. Long short-term memory (LSTM) networks are well suited for this task, as they can capture longitudinal variations in anticoagulation parameters and analyze historical sequences to support long-term dose prediction when integrated into clinical software systems (Wang et al., 2024). In external validation, the LSTM model achieved an INR prediction accuracy of 80.2% but only 66.6% accuracy for dose prediction, which was not substantially better than conventional models. To further enhance temporal feature extraction, we introduced a bidirectional structure and developed the Bi-LSTM dose prediction model. The final Bi-LSTM model achieved a dose prediction accuracy of 80.3% and a stable-dose prediction accuracy of 93.2% in external validation, demonstrating the feasibility of improving predictive performance through more comprehensive time-series modeling.

Regarding prediction errors, the Bi-LSTM model reduced the rate of overdosing predictions for actual doses to 11.9%, which was significantly lower than that of the LSTM model (*P* < 0.001). Considering that Asian populations are generally more sensitive to warfarin and require lower therapeutic doses, overdosing poses greater clinical risks than underdosing. Therefore, this reduction in overdosing errors highlights the improved safety of the Bi-LSTM approach. Subgroup analyses based on genotype showed that both models performed best in patients with normal warfarin response. In the sensitive subgroup, the Bi-LSTM model showed a modest decline in predictive accuracy, but the difference was not statistically significant, suggesting that it remains robust and applicable across different genotypes.

From the perspective of longitudinal follow-up, both models demonstrated their lowest prediction accuracy at the second visit, which may be attributed to the delayed pharmacodynamic onset of warfarin and the pronounced INR fluctuations observed within 3–5 days after initiation. This instability may contribute to increased prediction errors in the early treatment phase. During the first four visits, the Bi-LSTM model achieved slightly higher accuracy than the LSTM model (*P* > 0.05), but from the fourth visit onward, its advantage became statistically significant, indicating stronger capacity for temporal feature extraction in long sequences.

Genotype is a critical variable for warfarin dose prediction, and the absence of this information led to performance declines in both models. Notably, however, the Bi-LSTM model maintained relatively high predictive accuracy (78.7% for actual dose and 90.1% for stable dose). Consistent with previous findings, we observed that genetic polymorphisms exert a greater influence during the initiation phase than in the maintenance phase of warfarin therapy. Thus, genetic testing is particularly valuable in the early treatment period, whereas in long-term treatment, clinical factors and time-series data alone may provide sufficient information for reliable dose prediction. Compared with conventional models and unidirectional LSTM models, the Bi-LSTM model demonstrated superior practicality and adaptability, particularly in primary care settings or when genotype data are unavailable.

Compared with LSTM, the Bi-LSTM dosing model boasts strong capabilities in extracting temporal features and capturing data correlations, making it a reliable tool for warfarin dose prediction. Meanwhile, its in-depth utilization of time-series data can be well integrated with the systematic management functions of the AI-WAR software. Through deep integration with the anticoagulation data management system, the Bi-LSTM dosing model can conduct refined analysis of dynamic data generated during patients’use of the software, and adjust their medication doses timely and effectively. This thereby achieves more accurate and effective individualized warfarin dosing monitoring and management, and improves the efficacy and safety of warfarin therapy. Moreover, the advantages of Bi-LSTM have been demonstrated in other clinical domains involving complex time-series data, such as epilepsy and cardiovascular diseases (Zhu et al., 2019; Cao et al., 2025), further supporting its robustness and generalizability in healthcare applications.

This study has certain limitations. First, all participants were recruited from Hunan Province, China. Although the population structure in this region is largely comparable to that of the overall Chinese population, and the study sample to some extent represents Chinese warfarin users, the generalizability of the findings still requires further validation in larger, multicenter cohorts (Guyatt et al., 2011). Second, the modeling data were mainly derived from a randomized controlled trial (XY3-WAR study, n = 624), in which patients were followed up at regular intervals. This differs from the irregular monitoring that is common in real-world practice. To enhance the generalizability of the model, we additionally incorporated data from a prospective registry study (n = 176) and further performed external validation using real-world data from a prospective cohort study (n = 200), in which INR monitoring was carried out irregularly. Despite these limitations, we believe that the model performance assessment demonstrates good real-world reliability.

## 5 Conclusion

We developed and externally validated an AI-enabled warfarin decision-support system that couples an anticoagulation management platform with individualized dosing prediction models. Deployment of the software was associated with meaningful gains in clinical effectiveness and long-term safety. While the LSTM-based INR predictor achieved strong accuracy, a Bi-LSTM dose model further improved dosing performance over a unidirectional LSTM, underscoring the value of richer temporal feature extraction for individualized therapy. The system is practical for primary care settings with limited specialist resources, facilitating the translation of precision anticoagulation into routine practice and reducing clinician workload. Future work will integrate patient-generated health data and evaluate generalizability across broader populations and additional anticoagulants in multicenter prospective studies.

## Data Availability

The raw data supporting the conclusions of this article will be made available by the authors, without undue reservation.

## Supplementary material

Supplementary Figure S1 Trend of loss function with epoch of Bi-LSTM dose model.

Supplementary Figure S2 Trend of loss function with epoch of LSTM dose model.
