# Supplementary figures and images for: AI-WAR: a novel warfarin management software with a bidirectional LSTM dosing model improves time in therapeutic range
Source: Front Pharmacol. 2026 Apr 29;17:1750503. doi: 10.3389/fphar.2026.1750503 (PMC13167945; doi:10.3389/fphar.2026.1750503)

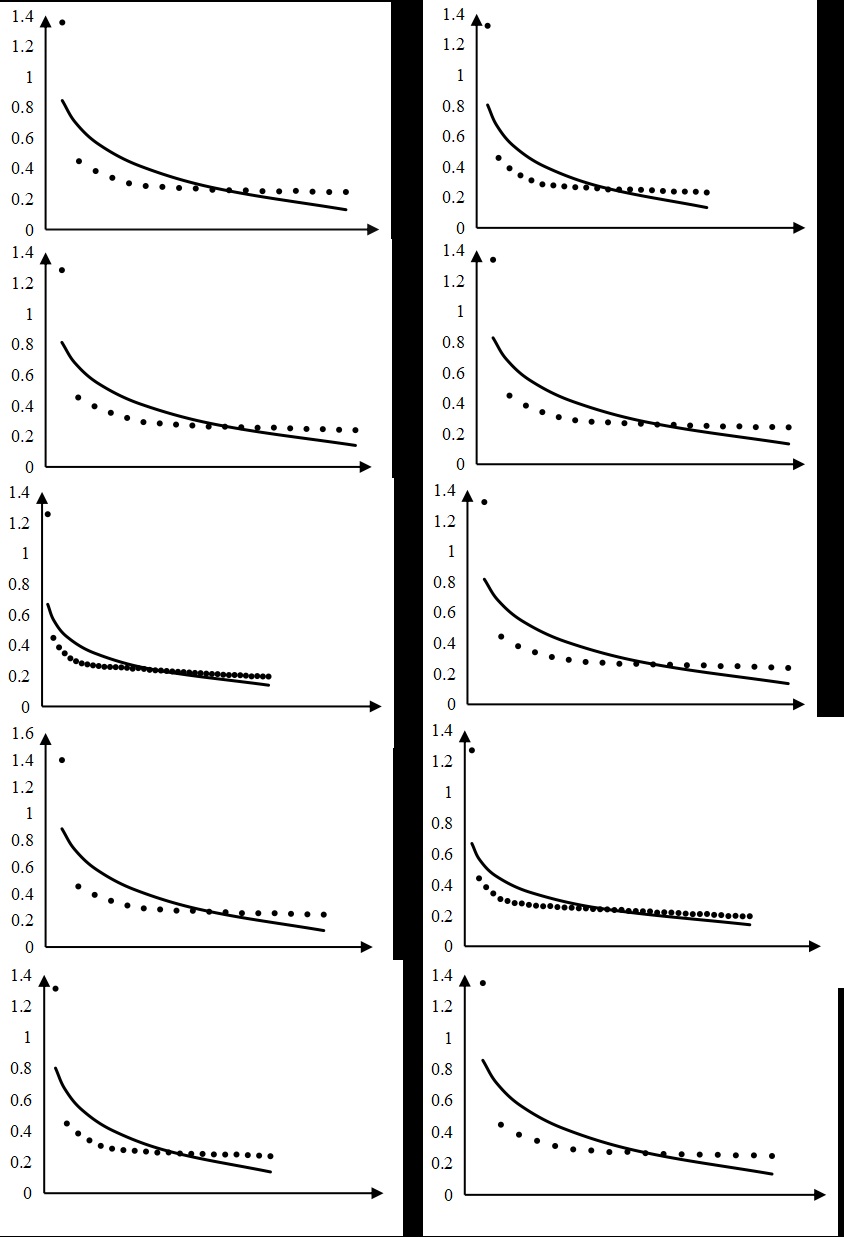

Supplement: Supplementary file 1 [file Image2.jpg]

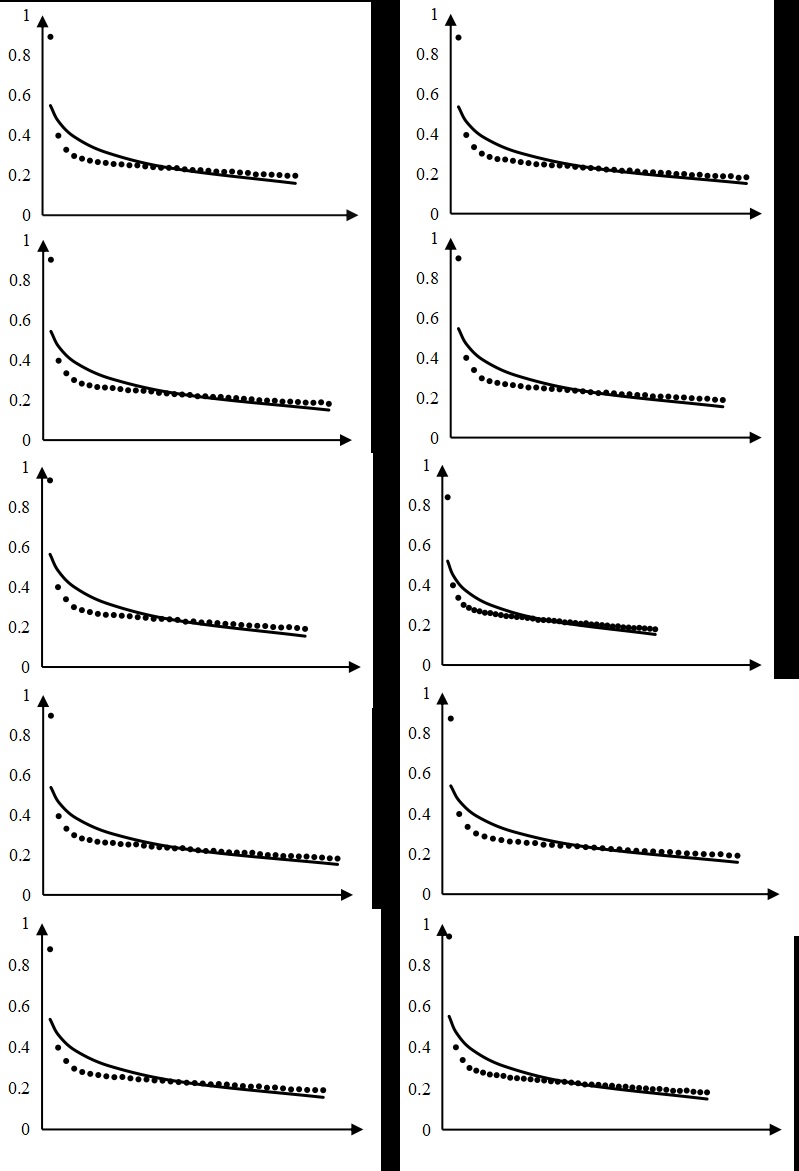

Supplement: Supplementary file 3 [file Image1.jpg]
