# Supplementary material for: AI-WAR: a novel warfarin management software with a bidirectional LSTM dosing model improves time in therapeutic range
Source: Front Pharmacol. 2026 Apr 29;17:1750503. doi: 10.3389/fphar.2026.1750503 (PMC13167945; doi:10.3389/fphar.2026.1750503)
Supplement: Supplementary file 2 [file Supplementaryfile1.docx]

Supplementary Material

# Supplementary Figures and Tables

## Supplementary Figures

**Supplementary Figure 1.** Trend of loss function with epoch of Bi-LSTM dose model

**Supplementary Figure 2.**Fig S2 Trend of loss function with epoch of LSTM dose model

## Supplementary Tables

Table S1 Demographic and clinical information in three clinical studies

| Characteristics | XY3-WAR  RCT study  （n=624） | Registration study（n=176） | AI-WAR  Test Group  （n=200） |
| --- | --- | --- | --- |
| Male, n (%) | 316（50.6） | 90（51.1） | 90 (45.0) |
| Age（y） | 67.4±10.1 | 53.2±10.0 | 55.7±10.3 |
| Height (cm) | 161.8±8.1 | 161.2±7.6 | 160.7±8.2 |
| Weight (kg) | 62.0±12.2 | 62.5±15.9 | 58.8±11.0 |
| Initial dose（mg） | 2.5±0.5 | 2.7±0.8 | 2.5±0.7 |
| Baseline INR | 1.0±0.1 | 1.4±0.5 | 1.5±0.5 |
| Indication, n (%) |  |  |  |
| Atrial fibrillation | 541（86.7） | 28（15.9） | 6（3.0） |
| Deep vein thrombosis | 83（13.3） | 8 (4.5) | 0 |
| Heart valve replacement | 0 | 167 (94.9) | 151（75.5） |
| Heart valve repair | 0 | 139 (79.0) | 173 (86.5) |
| Combined disease/drug use, n (%) | |  |  |
| Hypertension | 319（51.1） | 36（20.5） | 46 (23.0) |
| Diabetes | 207（33.2） | 10（5.7） | 12 (6.0) |
| Amiodarone | 9（1.4） | 2（1.1） | 6 (3.0) |
| Digoxin | 102（16.3） | 149（84.7） | 176 (88.0) |
| *VKORC1* genotype, n (%) | |  |  |
| A/A | 500（80.1） | 132（84.6） | 161（80.9） |
| A/G | 113（18.1） | 24（15.4） | 37（18.6） |
| G/G | 11（1.8） | 0 | 1（0.05） |
| *CYP2C9* genotype, n (%) | |  |  |
| *1/*1 | 579（92.8） | 141（90.4） | 178（89.4） |
| *1/*3 | 43（6.9） | 15（9.6） | 21（10.6） |
| *3/*3 | 2（0.3） | 0 | 0 |
| Note: The RCT excluded 36 patients who withdrew from the study on day 4/5, resulting in 4578 structured data points from 624 patients; the registry study excluded patients missing baseline INR data or with fewer than three follow-up data points, leaving 709 structured data points from 176 patients. Missing genotype data in 20 patients in the registry study and 1 patient in the test group. | | | |

Table S2 Adverse events during follow-up

|  | Before Matching | | *P* | After Matching | | *P* |
| --- | --- | --- | --- | --- | --- | --- |
|  | Test Group  (n=200) | Control Group(n=200) |  | Test Group  (n=118) | Control Group(n=118) |  |
| Gingival bleeding | 11 | 20 | 0.092 | 10 | 14 | 0.400 |
| Nosebleed | 5 | 12 | 0.083 | 3 | 9 | 0.079 |
| Gastrointestinal hemorrhage | 10 | 12 | 0.661 | 7 | 7 | 1.000 |
| Skin ecchymosis | 5 | 20 | 0.002 | 1 | 12 | 0.002 |
| Cerebral hemorrhage | 2 | 4 | 0.411 | 2 | 2 | 1.000 |
| Total | 27 (13.5%) | 57 (28.5%) | <0.001 | 18 (9.6%) | 37 (19.7%) | <0.001 |

Table S3 Stability evaluation index of Bi-LSTM dose model for ten repeated modeling

| Times | MSE | RMSE | MAE | R^2^ | Accuracy rate (%) |
| --- | --- | --- | --- | --- | --- |
| 1 | 0.302 | 0.550 | 0.350 | 0.718 | 80.2 |
| 2 | 0.305 | 0.552 | 0.353 | 0.716 | 80.0 |
| 3 | 0.302 | 0.550 | 0.357 | 0.718 | 80.2 |
| 4 | 0.298 | 0.546 | 0.350 | 0.722 | 80.7 |
| 5 | 0.302 | 0.549 | 0.358 | 0.728 | 80.5 |
| 6 | 0.291 | 0.540 | 0.346 | 0.721 | 80.3 |
| 7 | 0.299 | 0.546 | 0.347 | 0.717 | 79.4 |
| 8 | 0.304 | 0.551 | 0.355 | 0.710 | 80.3 |
| 9 | 0.318 | 0.564 | 0.362 | 0.716 | 79.0 |
| 10 | 0.302 | 0.550 | 0.350 | 0.718 | 80.2 |
| mean | 0.302 | 0.550 | 0.353 | 0.719 | 80.1 |
| SD | 0.007 | 0.006 | 0.005 | 0.005 | 0.5 |
| CV | 0.022 | 0.011 | 0.015 | 0.007 | 0.6 |

Table S4 Predictive performance of Bi-LSTM dose prediction model in external validation

| Times | Predicting True Dose | | | Predicting Stable Dose | | |
| --- | --- | --- | --- | --- | --- | --- |
|  | Accurate(%) | High(%) | Low(%) | Accurate(%) | High(%) | Low(%) |
| 1 | 78.6 | 15.6 | 5.8 | 90.9 | 8.4 | 0.8 |
| 2 | 79.6 | 14.8 | 5.7 | 90.9 | 8.2 | 0.9 |
| 3 | 78.5 | 14.9 | 6.6 | 91.0 | 7.5 | 1.5 |
| 4 | 79.1 | 14.5 | 6.4 | 91.2 | 7.5 | 1.4 |
| 5 | 78.6 | 14.9 | 6.5 | 90.7 | 8.1 | 1.2 |
| 6 | 79.1 | 14.6 | 6.3 | 89.6 | 8.8 | 1.5 |
| 7 | 79.0 | 14.2 | 6.8 | 89.8 | 8.1 | 2.1 |
| 8 | 79.6 | 14.1 | 6.3 | 89.8 | 8.2 | 2.0 |
| 9 | 79.0 | 14.1 | 6.8 | 90.4 | 8.2 | 1.4 |
| 10（Final model） | 80.3 | 11.9 | 7.8 | 93.2 | 5.6 | 1.2 |
| mean | 79.1 | 14.4 | 6.5 | 90.7 | 7.9 | 1.4 |
| SD | 0.6 | 1.0 | 0.6 | 1.0 | 0.9 | 0.4 |
| CV | 0.0 | 0.1 | 0.1 | 0.0 | 0.1 | 0.3 |

Table S5 Stability evaluation index of LSTM dose model for ten repeated modeling

| Times | MSE | RMSE | MAE | R^2^ | Accuracy rate (%) |
| --- | --- | --- | --- | --- | --- |
| 1 | 0.347 | 0.589 | 0.404 | 0.695 | 73.2 |
| 2 | 0.343 | 0.586 | 0.403 | 0.698 | 71.8 |
| 3 | 0.354 | 0.595 | 0.407 | 0.688 | 71.6 |
| 4 | 0.363 | 0.602 | 0.416 | 0.681 | 69.9 |
| 5 | 0.341 | 0.584 | 0.399 | 0.700 | 71.5 |
| 6 | 0.357 | 0.597 | 0.411 | 0.687 | 70.5 |
| 7 | 0.357 | 0.598 | 0.413 | 0.686 | 70.3 |
| 8 | 0.334 | 0.578 | 0.386 | 0.706 | 72.6 |
| 9 | 0.347 | 0.589 | 0.405 | 0.695 | 71.8 |
| 10 | 0.417 | 0.608 | 0.369 | 0.675 | 71.5 |
| mean | 0.356 | 0.593 | 0.402 | 0.691 | 71.5 |
| SD | 0.023 | 0.009 | 0.014 | 0.009 | 1.0 |
| CV | 0.065 | 0.015 | 0.035 | 0.014 | 1.3 |

Table S6 Comparison results of optimal predictive performance between two dose prediction models in external validation

| model | Predicting True Dose | | | | Predicting Stable Dose | | | |
| --- | --- | --- | --- | --- | --- | --- | --- | --- |
|  | Accurate(%) | High(%) | | Low(%) | Accurate(%) | High(%) | | Low(%) |
| Bi-LSTM | 80.3 | 11.9 | 7.8 | | 93.2 | 5.6 | 1.2 | |
| LSTM | 72.4 | 23.2 | 4.4 | | 84.0 | 15.5 | 0.5 | |
| *p* | <0.001 | <0.001 | <0.001 | | <0.001 | <0.001 | 0.226 | |

Table S7. Comparison of absolute dose errors between Bi-LSTM and LSTM models with respect to true and stable doses

| Outcome | Model | Median (IQR), mg/day | | | |
| --- | --- | --- | --- | --- | --- |
|  |  | total | <2mg/day | 2~3mg/day | >3mg/day |
| True Dose | Bi-LSTM | 0.22(0.10~0.41) | 0.23(0.10~0.44) | 0.17(0.08~0.32) | 0.30(0.14~0.58) |
|  | LSTM | 0.29(0.12~0.49) | 0.33(0.17~0.61) | 0.23(0.10~0.39) | 0.32(0.14~0.54) |
| Stable dose | Bi-LSTM | 0.16(0.08~0.29) | 0.14(0.08~0.26) | 0.15(0.07~0.25) | 0.22(0.11~0.35) |
|  | LSTM | 0.20(0.10~0.36) | 0.29(0.15~0.42) | 0.16(0.07~0.30) | 0.24(0.11~0.45) |

Table S8 Genotype subgroup

| Groups | Genotypes | Modeling data set | External verification set |
| --- | --- | --- | --- |
| Normal reaction subgroup | *CYP2C9**1/*1 and *VKORC1* GG/GA | 710 | 177 |
| Sensitive reaction subgroup | *CYP2C9**1/*1 and *VKORC1* AA，*CYP2C9**1/*3 and *VKORC1* GG/GA | 87 | 22 |
| Highly sensitive subgroup | *CYP2C9**1/*3 and *VKORC1* AA，*CYP2C9**3/*3 and *VKORC1* GG/GA/AA | 3 | 0 |

Table S9 Effect of genotype on the performance of two models

| Model | Conditions | Predicting True Dose | | | Predicting Stable Dose | | |
| --- | --- | --- | --- | --- | --- | --- | --- |
|  |  | Accurate(%) | High(%) | Low(%) | Accurate(%) | High(%) | Low(%) |
| Bi-LSTM | Normal reaction | 80.8 | 12.2 | 7.0 | 93.2 | 5.9 | 0.9 |
|  | Sensitive reaction | 76.2 | 10.4 | 13.5 | 92.9 | 3.6 | 3.6 |
|  | *P* | 0.129 | 0.457 | 0.002 | 0.909 | 0.533 | 0.116 |
|  | With genotype | 80.3 | 11.9 | 7.8 | 93.2 | 5.6 | 1.2 |
|  | Without genotype | 78.7 | 13.4 | 7.9 | 90.1 | 9.6 | 0.3 |
|  | *P* | 0.240 | 0.186 | 0.899 | 0.046 | 0.007 | 0.057 |
| LSTM | Normal reaction | 73.9 | 21.4 | 4.7 | 88.5 | 11.0 | 0.5 |
|  | Sensitive reaction | 63.2 | 35.2 | 1.6 | 53.6 | 46.4 | 0.0 |
|  | *P* | 0.002 | <0.001 | 0.042 | <0.001 | <0.001 | 1.000 |
|  | With genotype | 72.4 | 23.2 | 4.4 | 84.0 | 15.5 | 0.5 |
|  | Without genotype | 68.6 | 28.0 | 3.4 | 73.7 | 25.7 | 0.6 |
|  | *P* | 0.013 | 0.001 | 0.136 | <0.001 | <0.001 | 1.000 |
